# 

**Published:** 1901-05-15

**Authors:** 


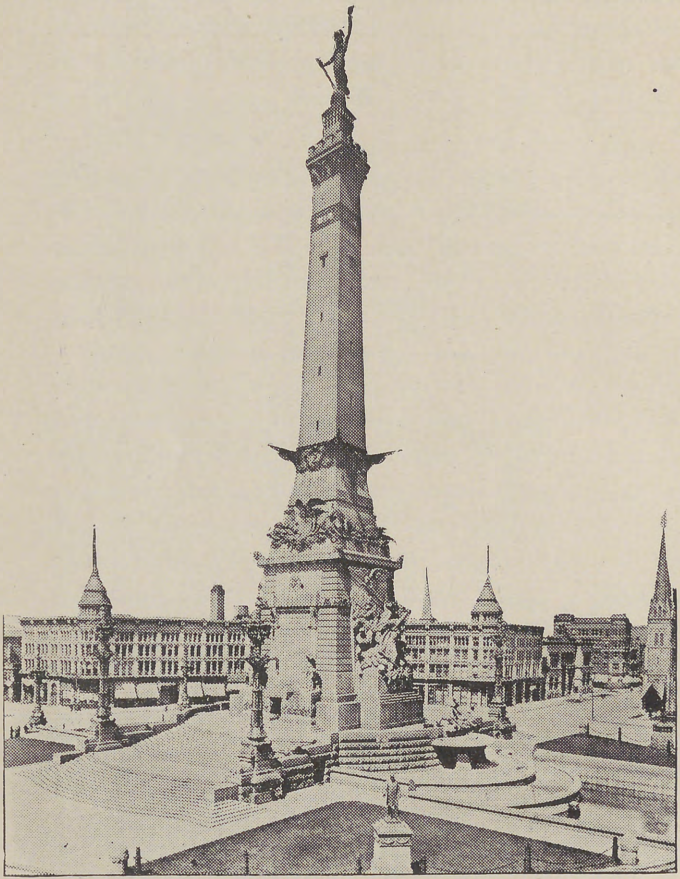

INDIANA'S SOLDIERS AND SAILORS MONUMENT.

### PROGRAM OF INFORMATION FOR TRI-STATE DENTAL MEETING.

This year Indiana is to entertain Ohio and Michigan at Indianapolis, June 4th, 5th, 6th. The meeting, clinics and exhibits will be held at the German House, a private club house on the southeast corner of Michigan and New Jersey streets.

### RAILROAD INFORMATION.

The Central Passenger Association has granted a round trip rate of one and one-third fares on the certificate plan. When you buy your ticket to Indianapolis, pay full fare, and secure a certificate from the ticket agent that you are coming to the Tri-State Dental Meeting. The first time you go to the German House, surrender your certificate to the clerk, register, receive a check for your certificate and a button. The agent for the Passenger Association will be on hand on Wednesday and Thursday, to vise certificates. You can secure your certificate on surrendering your check any time after Wednesday evening, provided you handed it in promptly on your arrival in the city. Your certificate will entitle you to purchase a return trip ticket for one-third of the fare paid in coming. Tickets on the certificate plan can be purchased at any ticket office in the Central Passenger Association's territory for trains which leave between midnight of Friday, May 30th, to midnight of Wednesday, June 5th. Return tickets can be purchased at Indianapolis for trains which leave before midnight of Monday, June 10th. All trains arrive at and depart from the Union Station.

### FROM UNION STATION TO HOTELS.

Cabs and carriages may be had at the station. The fare to the hotels is twenty-five cents for each person.

The transfer charge for trunks is twenty-five cents each. The Denison House may be reached by the following car lines, all of which pass the Union Station: College Avenue, Central Avenue, Pennsylvania Street, Alabama Street and East Tenth Street. The Bates House may be reached on all cars running north on Illinois Street. English Hotel may be reached by taking an Illinois street car to Market, thence east to Monument Place. The Grand and the Spencer are both close to the Union Station.

HOTEL RATES.

Denison House and Bates House, $3.00 and up; English Hotel and Grand Hotel, $2.50 and up; Spencer House, $2.00 and up.

STREET CAR FACTS.

Cash fare, five cents; six tickets are sold for twenty-five cents; twenty-five tickets for $1.00. Transfers from any line to any other line may be had if application is made to the conductor at the time fare is paid. All College Avenue, East Tenth and Broad Ripple cars pass the German House.

LADIES.

Important! All visiting ladies are requested to meet the Ladies' Committee in the parlors of the Denison House at 3:00 P. M. on Tuesday.

All visitors who expect to bring ladies with them are urged to notify George E. Hunt, 131 E. Ohio Street, Indianapolis, some time before the meeting.

PROGRAM—TUESDAY, JUNE 4TH.

Morning, 10 o'clock—Papers and Discussion.
Afternoon, 2 o'clock—Papers and Discussion.
Evening, 8 o'clock—Papers and Discussion.

WEDNESDAY, JUNE 5TH.

Morning, 9 o'clock—Clinics.

Afternoon, 2 o'clock—Papers and Discussion.
Evening, 8 o'clock—Reception and Band Concert.

THURSDAY, JUNE 6TH.

Morning, 9 o'clock—Papers and Discussion.
Afternoon, 2 o'clock—Papers and Discussion.
Evening, 8 o'clock—Papers and Discussion.

PAPERS.

NOTE.—The papers will be presented in the order which seems to serve the best interests of the meeting.

1. "Critical Periods in the History of the Human Teeth." By C. N. Johnson, L.D.S., D.D.S., Chicago, Ills.

Discussion opened by H. A. Smith, Cincinnati, Ohio, and H. B. Tilleston, Louisville, Ky.

2. "Porcelain as an Art in Dental Work." By Fred'k J. Capon, M.D.S., D.D.S., Toronto, Canada.

Discussion opened by John E. Nyman, Chicago, Ills., and Hart J. Goslee, Chicago, Ills.

3. "Continued Anesthesia with Nitrous Oxid Gas under Definite Pressure." By W. A. Heckard, D.D.S., Indianapolis, Ind.

Discussion opened by O. N. Heise, Cincinnati, Ohio.

4. "Some Considerations in Modern Bridge Work." By J. L. Young, D.D.S., Detroit, Mich.

Discussion opened by W. W. Munger, Ft. Wayne, Ind., and J. F. Stephan, Cleveland, Ohio.

5. "Dental Neurasthenia." By W. H. Whitslar, M.D., D.D.S., Cleveland, Ohio.

Discussion opened by R. I. Blakeman, Indianapolis, Ind., and E. T. Loeffler, Saginaw, Mich.

6. "Diseases of the Gums and Sockets of the Alveoli." By C. M. Wright, M.D., D.D.S., Cincinnati, Ohio.

Discussion opened by J. E. Cravens, Indianapolis, Ind., and J. Taft, Ann Arbor, Mich.

7. "A Study of Tooth Bleaching." By N. S. Hoff, D.D.S., Ann Arbor, Mich.

Discussion opened by J. S. Cassidy, Covington, Ky., and J. P. Buckley, Chicago, Ills.

8. "The Human Face and Articulate Speech Divine." By George E. Johnson, D.D.S., Ft. Wayne, Ind.

Discussion opened by Grant Molyneaux, Cincinnati, Ohio.

LECTURES AND TALKS.

1. Weston A. Price, D.D.S., Cleveland, Ohio.—"Application of Roentgen Rays in Dentistry."

2. C. S. Case. M.D., D.D.S., Chicago, Ills.—"Correction of Dento-Facial Deformities," illustrated with the lantern.

3. W. E. Griswold, D.D.S., Denver, Colo.—"A System of Removable Bridges."

4. Truman W. Brophy, Chicago, Ills.—"Stereopticon Exhibit on Surgery of the Palate."

CLINICS.

1. J. W. Clark, Louisville, Ky.—"Combination Amalgam and Gold Filling."

2. W. E. Grant, Louisville, Ky.—"Some Appliances Used in Orthodontia."

3. J. J. Wright, Milwaukee, Wis.—"An Appliance for Keeping the Field Dry While Setting Crowns and Bridges."

4. W. E. Harper, Chicago, Ills.—"Exhibit of Prepared Cavities, with Practical Suggestions."

5. R. B. Tuller, Chicago, Ills.—"Some Suggestions About the Use of Matrices."

6. J. P. Carmichael, Milwaukee, Wis.—"Abutment Piece and a Support in Extensive Gold and Porcelain Inlays."

7. J. E. Nyman, Chicago, Ills.—"Carved Solid Cusp for Gold Crowns."

8. George B. Perry, Chicago, Ills.—"A Method of Mounting and Truing Wheels for Engine and Lathe."

9. H. J. Goslee, Chicago, Ills.—"Porcelain Crown and Bridge Work."

10. E. J. Perry, Chicago, Ills.—"Prosthetic Wrinkles."

11. C. V. Vignes, New Orleans, La.—"Extracting Forceps with Removable Beaks, and a New Amalgam Carrier."

12. Harry P. Carlton, San Francisco, Cal.—"Use of Knowles' Pluggers in Matrix Work, and One Kind of Matrix."

13. F. J. Capon, Toronto, Canada.—"Porcelain Inlays."

14. C. E. Esterly, Lawrence, Kans.—"Blowpipe for Compressed Air, and Modified Davidson Spray."

15. George E. Hunt, Indianapolis, Ind.—"A Scientific Instrument for Measuring Bulk Changes in Plastics."

16. F. M. Bozer, Logansport, Ind.—"Comparative Anatomy Exhibit."

17. F. R. McClanahan, Rushville, Ind.—"Possibilities of the Dental Plate."

18. H. A. Moyer, Kendallville, Ind.—"Richmond Crown with Soldered Fillings."

19. E. E. Reese, Indianapolis, Ind.—"Porcelain Inlays."

20. D. A. House, Indianapolis, Ind.—"Porcelain Crowns."

21. C. E. Redmon, Peru, Ind. — "Porcelain Crowns."

22. W. O. Vallette, Goshen, Ind.—"Practical Ideas in Crown and Bridge Work."

23. H. L. Ambler, Cleveland, Ohio.—"Filling Teeth with Improved Tin Foil."

24. C. R. Butler, Cleveland, Ohio.—"Adaptability of a New Form of Scalers."

25. E. Ballard Lodge, Cleveland, Ohio.—"Matrix and Matrix Plugger."

26. S. D. Ruggles, Portsmouth, Ohio.—"The Removal of Excess Cement in Setting Crowns."

27. Blair Blackmarr, Jackson, Mich.—To be announced.

28. Rudolph Beck, Chicago, Ills.—"Adjustment of a Gold Crown with a Special Instrument Designed by the Clinician."

29. J. L. Sweetnam, Manistee, Mich.—"Something New in Crown Work."

30. L. F. Owen, Grand Rapids, Mich.—"Gold Inlays."

31. H. D. Watson, Grand Rapids, Mich.—"Seamless Platinum Cup for Porcelain Work."

34. D. Miller, Chicago, Ills.—"Contouring Pliers."

35. F. W. Stephan, Chicago, Ills.—"Use of the Matrix and Pad."

36. J. O. Stillson and W. A. Heckard, Indianapolis.—"Removal of an Eye Under Continued Anesthesia." Dr. Stillson, specialist in eye and ear surgery, will perform the operation and Dr. Heckard will administer the anesthetic.

37. Jas. W. Cowan, Geneseo, N. Y.—"Floss Toothpick."

38. J. R. Bell, Cleveland, Ohio.—"Decay, and Saliva Ejector."

39. L. E. Custer, Dayton, Ohio. — "Improved Electric Furnace."

NOTE.—A complete list will be distributed at the meeting.

### ANNOUNCEMENTS.

### NEW JERSEY STATE DENTAL SOCIETY.

The meeting to be held at Asbury Park, N. J., July 17th to 19th, inclusive, promises to be the greatest meeting in the history of State Societies, from an educational standpoint. The applications already on file for exhibition space exceeds that of last year, insuring a display of dental appliances, etc., which will almost entirely fill the great Auditorium. The young graduates will do well to avail themselves of this opportunity and so plan their vacation as to combine business with pleasure and see all the very latest appliances which will be on exhibition. The "Hornets" wish it distinctly understood that the "latch string" will be upon the outside of the door during the meetings.

W. L. Fish, *Chairman.*

The Chicago Dental Society elected the following officers for the ensuing year April 2d: President, A. B. Clark; First Vice-President, G. B. Perry; Second Vice-President, B. D. Wikoff; Secretary, E. MaWhinney; Cor. Sec'y, C. S. Bigelow; Treasurer, E. R. Carpenter; Librarian, H. W. Sale; Member Board of Directors, J. G. Reid; Board of Censors, W. V. B. Ames, C. N. Johnson and A. W. Harlan.

---

The Southern Branch of the National Dental Association will meet July 29th at Nashville, Tenn.

C. L. Alexander, *Secretary*.

---

The Ohio State Board of Dental Examiners will meet Tuesday, May 28, 1901, in Columbus, Ohio. Applicants for examination should bring instruments for making gold fillings, except engine. Full particulars may be had by addressing

Dr. L. P. Bethel, *Secretary*,
Kent, Ohio.

---

The Missouri State Dental Society will meet July 9, 10, 11 and 12, 1901, at Sedalia, Mo. A cordial invitation is extended to all reputable practitioners.

B. L. Thorp, *Secretary*,
St. Louis, Mo.

---

Northern Ohio Dental Society.—On account of the Tri-State Meeting, there will be no meeting of this society until June, 1902.

W. T. Jackman, *Secretary*.

